# Enhancement of the chondrogenic differentiation capacity of human dental pulp stem cells via chondroitin sulfate-coated polycaprolactone-MWCNT nanofibers

**DOI:** 10.1038/s41598-024-66497-w

**Published:** 2024-07-16

**Authors:** Ghada Nour Eldeen, Tarek A. Elkhooly, Gehan T. El Bassyouni, Tamer M. Hamdy, Ahmed R. Hawash, Riham M. Aly

**Affiliations:** 1 Human Genetics and Genome Research Institute, National Research Centre, Dokki, Giza, 12622 Egypt; 2 Refractories, Ceramics, and Building Materials Department, National Research Centre, Dokki, Giza, 12622 Egypt; 3 Nanomedicine Research Unit, Faculty of Medicine, Delta University for Science and Technology, Gamasa, Egypt; 4 Restorative and Dental Materials Department, Oral and Dental Research Institute, National Research Centre, Dokki, Giza, 12622 Egypt; 5 Faculty of Medicine, Delta University for Science and Technology, Gamasa, Egypt; 6 Basic Dental Science Department, Oral and Dental Research Institute, National Research Centre, 33 El Bohouth St., Dokki, Giza, 12622 Egypt; 7 Stem Cells Lab, Center of Excellence for Advanced Sciences, National Research Centre, Dokki, Giza, 12622 Egypt

**Keywords:** Chondrogenic differentiation, PCL scaffolds, Human dental pulp stem cells, Regeneration, Chondroitin sulfate, Cell biology, Medical research, Stem-cell research

## Abstract

Most of the conditions involving cartilaginous tissues are irreversible and involve degenerative processes. The aim of the present study was to fabricate a biocompatible fibrous and film scaffolds using electrospinning and casting techniques to induce chondrogenic differentiation for possible application in cartilaginous tissue regeneration. Polycaprolactone (PCL) electrospun nanofibrous scaffolds and PCL film were fabricated and incorporated with multi-walled carbon nanotubes (MWCNTs). Thereafter, coating of chondroitin sulfate (CS) on the fibrous and film structures was applied to promote chondrogenic differentiation of human dental pulp stem cells (hDPSCs). First, the morphology, hydrophilicity and mechanical properties of the scaffolds were characterized by scanning electron microscopy (SEM), spectroscopic characterization, water contact angle measurements and tensile strength testing. Subsequently, the effects of the fabricated scaffolds on stimulating the proliferation of human dental pulp stem cells (hDPSCs) and inducing their chondrogenic differentiation were evaluated via electron microscopy, flow cytometry and RT‒PCR. The results of the study demonstrated that the different forms of the fabricated PCL-MWCNTs scaffolds analyzed demonstrated biocompatibility. The nanofilm structures demonstrated a higher rate of cellular proliferation, while the nanofibrous architecture of the scaffolds supported the cellular attachment and differentiation capacity of hDPSCs and was further enhanced with CS addition. In conclusion, the results of the present investigation highlighted the significance of this combination of parameters on the viability, proliferation and chondrogenic differentiation capacity of hDPSCs seeded on PCL-MWCNT scaffolds. This approach may be applied when designing PCL-based scaffolds for future cell-based therapeutic approaches developed for chondrogenic diseases.

## Introduction

Cartilaginous tissue disorders in the human body range from relatively simple conditions such as osteoarthritis to considerably more complex diseases^1^. Osteoarthritis of the temporomandibular joint, for instance, is a prevalent condition characterized by wear and degeneration of the articular cartilage^2^. Unfortunately, the majority of conditions involving cartilaginous tissues are irreversible and can progress to degenerative processes^3^. Currently, there is no established and efficient treatment available to fully regenerate the thickness of the articular cartilage. As a result, tissue engineering has emerged as an approach to generate the desired tissue by utilizing the trinity of an appropriate cell population, scaffold, and growth factors to restore the functionality of damaged tissues^4^. Mesenchymal stem cells are considered the cell of choice in the field of tissue engineering owing to their remarkable plasticity, proliferative ability, and relative ease of accessibility^5–7^ Stem cells derived from human dental pulp are particularly sought after as a mesenchymal cell source due to their easily accessible nature, absence of ethical concerns, and, above all else, their capacity to differentiate into numerous lineages, including osteogenic and chondrogenic lineages^8–11^. In addition, these cells possess unique characteristics that promote a regenerative environment, including minimal immunogenicity and the ability to modulate the immune system^12,13^.

A three-dimensional microenvironment that can induce chondrogenic differentiation is of utmost importance in this regard. Therefore, it is necessary to utilize both biocompatible and bioactive scaffolds that possess appropriate porosity to facilitate the flow of nutrients while at the same time being able to withstand physiological stresses until adequate tissue regeneration occurs.

Biodegradable and biocompatible polymers, whether natural or synthetic, have been proposed as potential candidates in the field of regenerative medicine. Polycaprolactone (PCL), a biodegradable conventional bioactive polymer that has received approval from the FDA due to its remarkable biocompatibility, has attracted considerable attention within the field of tissue engineering. However, the applicability of PCL has been limited due to its high hydrophobicity, prolonged degradation time, and inadequate mechanical properties^14^.

To overcome these limitations, various physical and chemical approaches can be used to modify polymer surfaces to enhance polymer-cell interactions. Hereby, nanoscale structure like bio glass, magnetic mesoporous bioactive glass (MMBG), alumina nanowire and MWCNTs were incorporated into the PCL and enhanced its mechanical properties and biological performance as well as overcome its limitations^15–17^. Khoroushi et al. investigated the effect of nano-bioglass on Polyhydroxybutyrate (PHB)/Chitosan nanofibers towards stem cells particularly stem cells from human exfoliated deciduous teeth (SHEDs) differentiation^16^. The results showed that incorporation of bio glass and chitosan improved the mechanical properties of the hydrophobic polyhydroxybutyrate polymer and its hydrophilicity and finally enhanced the differentiation potential of SHED into odontoblast-like cells. Alternatively, Mirmusavi et al., used the mature chondrocytes were seeded on the PCL/chitosan nanofibers with and without of MWCNTs embedded into the nanofibers^15^. The results revealed that incorporation of 0.5 wt% of MWCNTs of the nanofibers had a positive effect on the viability and proliferation of chondrocytes. However, Najafabadi et al. in 2023 shown that coating a layer of MWCNTs/chitosan improved the cell survival, growth and osteoblast-like differentiation^14^. This finding suggests that this approach can be employed in the field of bone tissue engineering. Multiwall carbon nanotubes (MWCNTs) consist of several single-walled carbon nanotubes (CNTs) arranged in concentric layers. Due to their reduced structural flaws, MWNCTs have been proposed for application in tissue engineering. It has been reported that the incorporation of carbon nanotubes (CNTs) into polymers can improve their structural and physiochemical properties, including increased strength, flexibility, and biocompatibility^18^. Additionally, CNTs can regulate gene expression for the purpose of tissue regeneration^19^.

Moreover, the scaffolds utilized must provide an extracellular matrix (ECM)-like environment to promote the proliferative chondrogenic differentiation ability of stem cells. This could be accomplished through chemical modification of the polymer surface to direct cell fate determination and the formation of chondrogenic tissue. An alternative method for regulating the biological characteristics of nanofibers is to manufacture nanofibers made from a combination of different polymers, such as polyesters for improved mechanical strength, combined with carbohydrate polymers to induce specific cellular reactions^17^. Chondroitin sulfate (CS), which belongs to the glycosaminoglycan (GAG) family, is an important component of the extracellular matrix (ECM) and is predominantly present in cartilage and bone. CS can modulate cartilage formation and reduce inflammation^20^.

It is therefore important to note that the application of stem cell-based therapies coupled with suitable scaffolds offers potential as an effective approach for treating cartilage disorders, including temporomandibular joint osteoarthritis (TMJ) osteoarthritis, and regenerating full cartilage thickness in contrast to the available treatment options.

Thus, the aim of the present study was to fabricate a biocompatible fibrous and film scaffold with optimum physical properties that can induce chondrogenic differentiation for possible application in cartilaginous tissue regeneration. We fabricated different topographical structures of PCL incorporated with 2 wt% MWCNTs using electrospinning techniques for electrospun nanofibrous scaffold and using casting technique for film structure. Subsequently, coating of chondroitin sulfate was applied to promote chondrogenic differentiation of hDPSCs. First, the morphology, hydrophilicity, and mechanical properties of the scaffolds were characterized by scanning electron microscopy (SEM), spectroscopic characterization, water contact angle measurements and tensile strength testing. Subsequently, the effects of the fabricated scaffolds on stimulating the proliferation of human dental pulp stem cells (hDPSCs) and inducing their chondrogenic differentiation were evaluated via electron microscopy, flow cytometry and RT‒PCR for the evaluation of chondrogenic differentiation markers. To the best of our knowledge, there is a lack of published literature investigating the collective impact of CS-coated PCL-MWCNTs scaffolds on hDPSCs, with a focus on their biocompatibility and capacity for chondrogenic differentiation induction.

## Results

### Morphology of the PCL-MWCNT scaffolds

**Figure 1:**
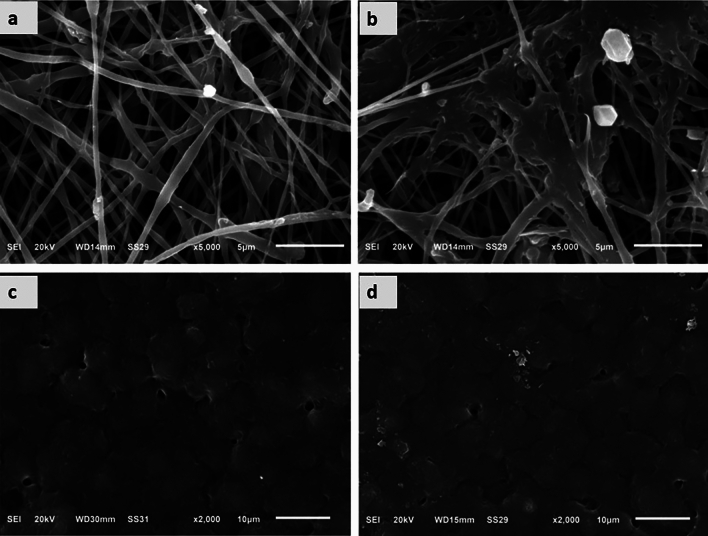
SEM microphotographs of nanofibrous and film PCL-MWCNT scaffolds with and without chondroitin sulfate (CS). (**a**) PCL-MWCNT nanofibers, (**b**) PCL-MWCNT nanofibers coated with CS, (**c**) PCL-MWCNT film, (**d**) PCL-MWCNT film coated with CS.

**Figure 2:**
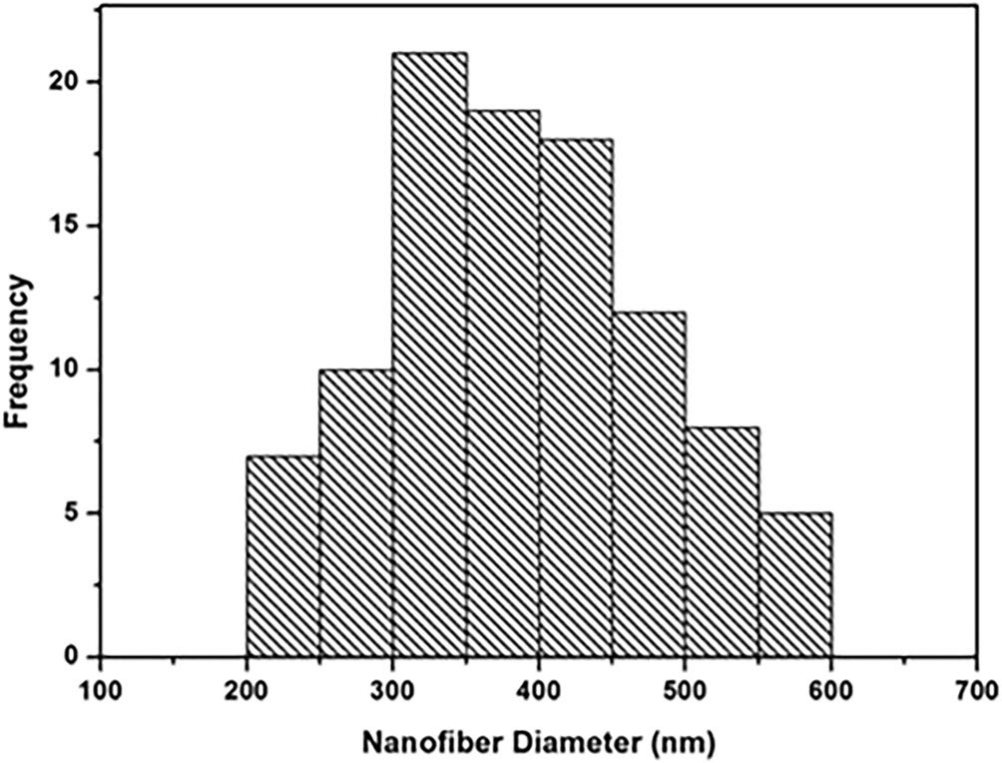
Histogram illustrating the range of PCL/MWCNTs nanofiber diameters. The average diameter was 386 ± 93 nm with a min. diameter of 211 nm and maximum diameter of 598 nm.

Figure 1 shows SEM micrographs of the electrospun PCL-MWCNTs scaffolds in the presence and absence of chondroitin sulfate (CS) coating. The PCL-MWCNTs nanofibers demonstrated a random orientation with a regular structure and a smooth surface without beads formation, and there was interconnected porosity between the nanofibers mesh. Minor amount of MWCNTs were observed on the nanofibers surface, indicating that most of the nanotubes were entrapped inside the nanofibers. PCL-MWCNTs nanofibers coated with chondroitin sulfate (CS) showed a random orientation with a regular structure and the presence of some granules, which may be attributed to the presence of CS. The size distribution analysis of the nanofiber’s diameters showed that the average diameter (Dav.) = 386 ± 93 nm with a min. diameter of 211 nm and maximum diameter of 598 nm (Fig. 2). For the morphological appearance of the PCL-MWCNTs films, a homogenous and relatively smooth surface with no pores in the revealed structure of the film was observed. Since functionalization was not conducted on the carbon nanotubes (i.e. carboxylation, amination, etc.), the casted films in figures C and D showed rough surface with a tendency of carbon nanotubes aggregation forming distinct areas like grain boundaries. Finally, PCL-MWCNTs films coated with CS exhibited the same morphology as those without CS coating implying less adsorbed quantity of CS on the surface in the case of film architecture.

### Contact angle analysis

**Figure 3:**
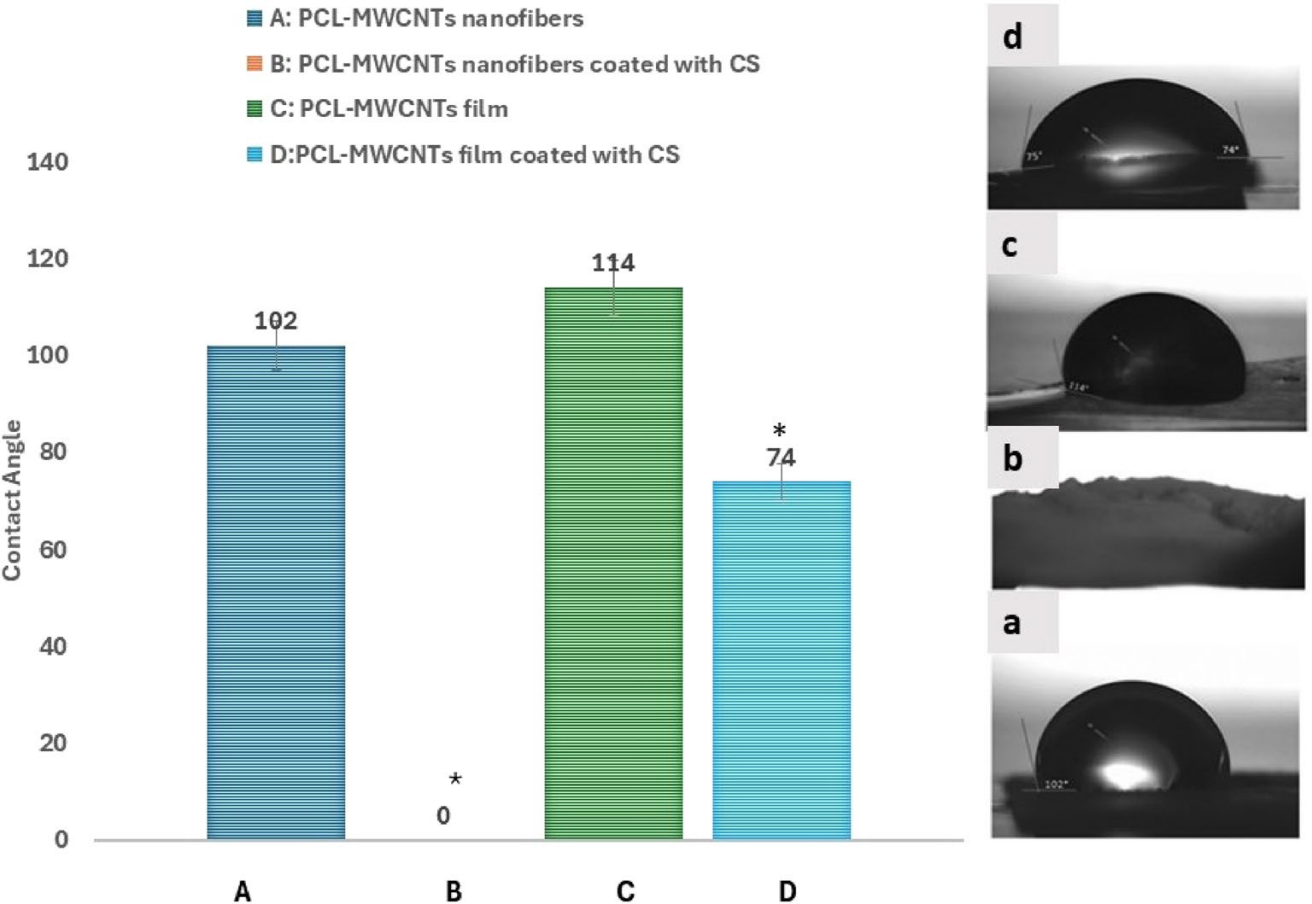
Water contact angle measurements of nanofibrous and film PCL-MWCNT scaffolds with and without CS. (**A**) PCL-MWCNT nanofibers, (**B**) PCL-MWCNT nanofibers coated with CS, (**C**) PCL-MWCNT film, (**D**) PCL-MWCNT film coated with CS. Group B showed the least contact angle (zero). This was followed by group D, and then group A. The highest contact angle (114) was displayed by group C (PCL-MWCNT film). A significant difference was found between the coated film or fibers with uncoated ones (*P = 0.0001).

The addition of CS induced hydrophilic properties in both the nanofibrous and film scaffolds, with a pronounced reduction in the contact angle. As illustrated in Fig. 3, more water was immediately absorbed into the fibrous network than into the film structure, resulting in a decrease in the contact angle. When comparing the contact angle of the film to the nanofibers without CS coating, the results were insignificant. However, when comparing the coated film or fibers with uncoated ones, the reduction in contact angle or the increase in surface wettability were significant (P = 0.0001). This might be attributed to the higher amount of absorbed hydrophilic CS on the nanofibrous structure when compared to the film structure.

### Young’s modulus analysis

**Figure 4:**
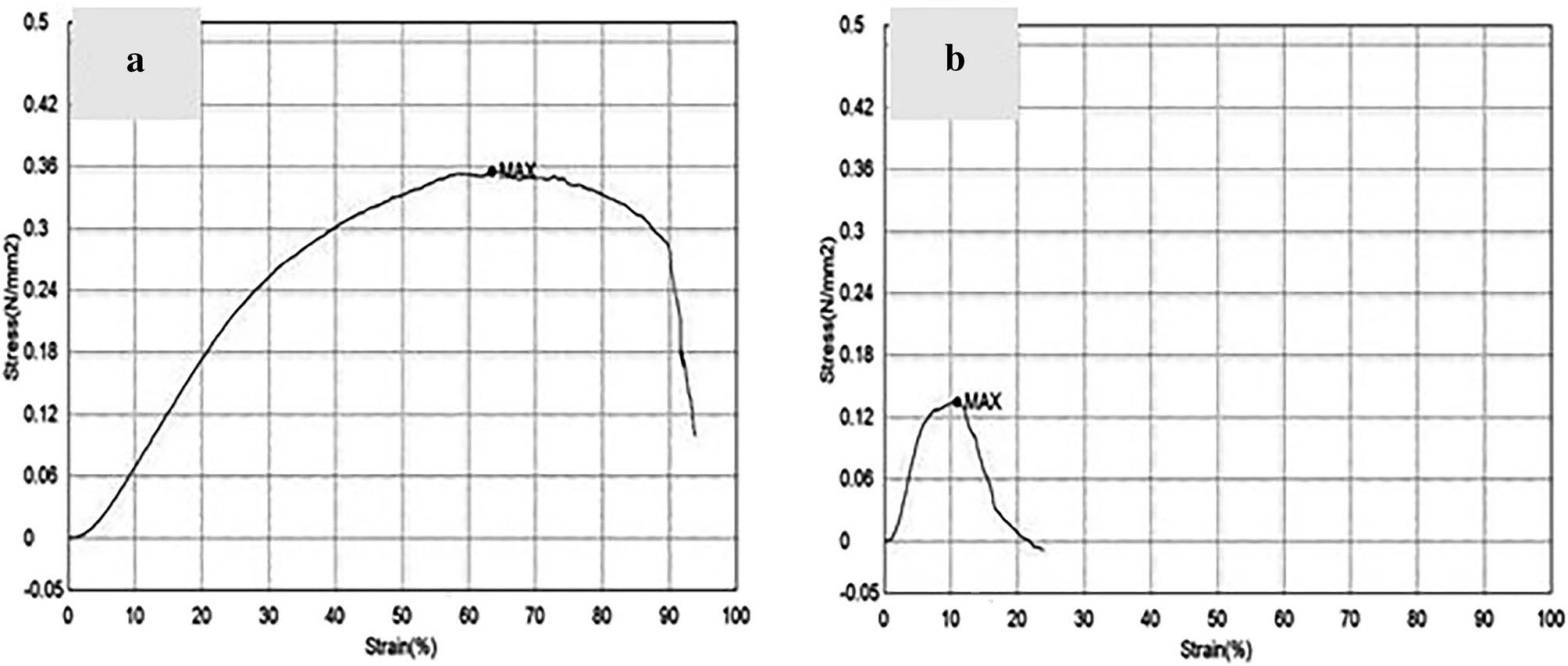
Stress–strain diagrams of PCL/MWCNTs nanofibers (**a**) and PCL/MCNTs film (**b**). The Young’s modulus of nanofibers (E = 108.19 ± 16.03 MPa) showed significantly higher value than the cast film (E = 29.30 ± 5.51 MPa). The Ultimate tensile strength (UTS) was 0.38 ± 0.07 and 0.15 ± 0.02 N/mm^2^ for nanofibers and film scaffolds, respectively.

Figure 4 illustrates stress–strain diagrams of PCL/MWCNTs nanofibers and PCL/MCNTs film. The Young’s modulus of nanofibers (E = 108.19 ± 16.03 MPa) showed significantly higher value than the cast film (E = 29.30 ± 5.51 MPa). The Ultimate tensile strength (UTS) was found to be 0.38 ± 0.07 and 0.15 ± 0.02 N/mm^2^ for nanofibers and film, respectively. While elongation at break (Eb) was found to be 59.62 ± 3.39 and 9.13 ± 1.38% for nanofibers and film, respectively.

### Surface roughness measurement

The roughness results (Table 1) demonstrated a significant increase in Ra and RMS value of nanofibers when compared to the film, while coating of CS didn’t influence roughness value in case of the film but it affected the roughness of the surface of the nanofibers leading to significant reduction in Ra and RMS values of the roughness.

**Table 1:** Surface roughness results of nanofibers when compared to the film in nanoscale.

Uncoated film		CS coated film	
Ra (nm)	RMS (nm)	Ra (nm)	RMS (nm)
46.87 ± 3.03	61.80 ± 4.62	42.24 ± 5.81	61.84 ± 11.86

Uncoated fibers		CS coated fibers	
Ra (nm)	RMS (nm)	Ra (nm)	RMS (nm)
182.61 ± 4.14	220.09 ± 5.08	112.74 ± 18.72	148.21 ± 19.49

### FTIR spectra analysis

**Figure 5:**
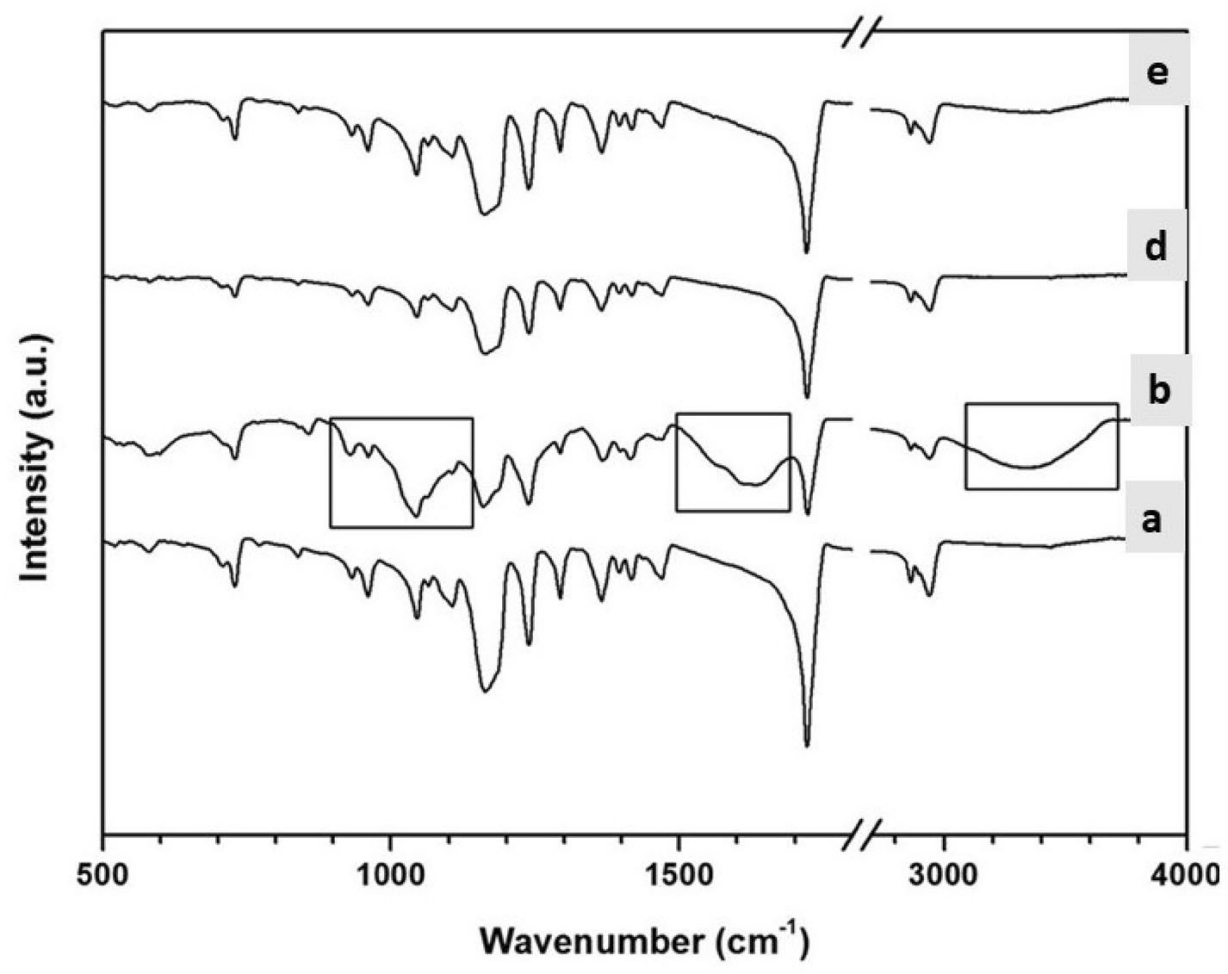
FTIR spectra of nanofibrous and film PCL-MWCNT scaffolds with and without chondroitin sulfate (CS). (**a**) PCL-MWCNT nanofibers, (**b**) PCL-MWCNT nanofibers coated with CS, (**c**) PCL-MWCNT film, (**d**) PCL-MWCNT film coated with CS.

FTIR spectroscopy was conducted to examine the distinctive absorption peaks of the chemical groups in the fabricated PCL-MWCNTs scaffolds, both in the film and nanofiber form (Fig. 5). The FTIR spectra of the PCL-MWCNTs film and nanofibers without the addition of CS were compared with those of the CS-coated PCL-MWCNTs film and nanofiber groups. Distinct absorption peaks were observed at 1652 cm^−1^ for the stretching vibration of C=O, 1542 cm^−1^ for the bending vibration of N–H, 1441 cm^−1^ for the stretching of CH2, and 1267 cm^−1^ for the stretching vibration of C–N. The CS spectra exhibited a distinct absorption band at 1245 cm^−1^, which corresponds to the stretching vibration of the S=O bond in the negatively charged SO4 ion. A comparison of the spectra of the PCL-MWCNTs- and CS-supplemented groups revealed that the CS-supplemented group exhibited a band characteristic of CS at 1245 cm^−1^. Additionally, the presence of CS in the nanofibers resulted in an increase in the intensity of the CS band compared to that of the film scaffolds. This finding confirmed the enhanced presence of CS in the electrospun PCL-MWCNTs nanofibers.

### Raman spectroscopy

**Figure 6:**
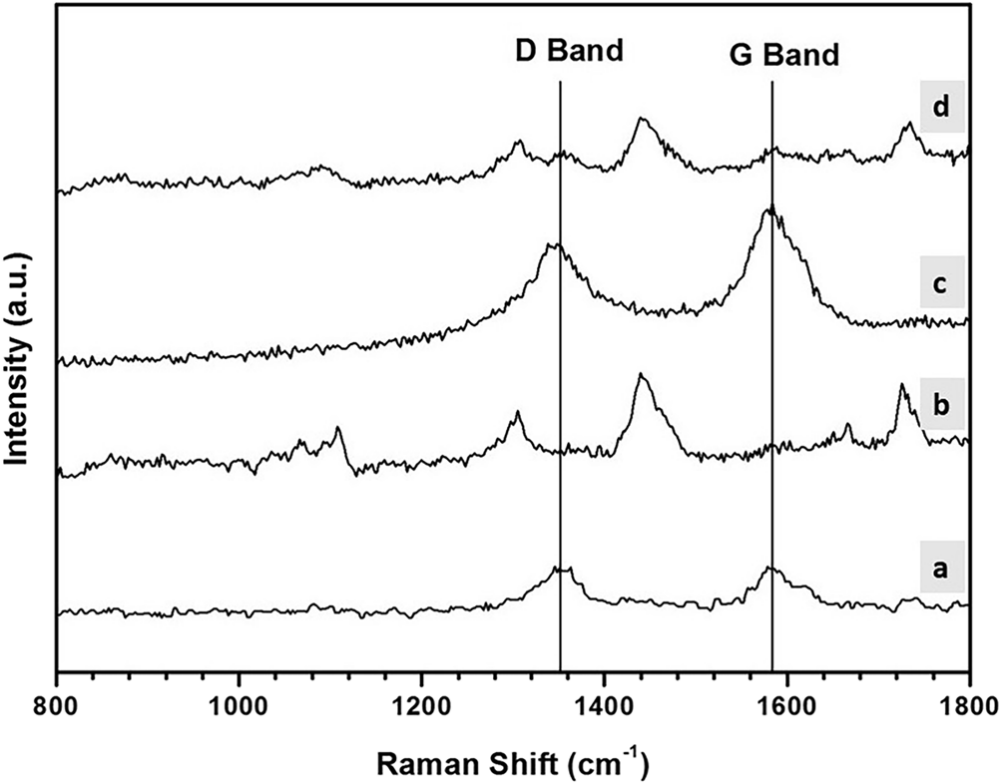
Raman spectra of nanofibrous and film PCL-MWCNT scaffolds with and without chondroitin sulfate (CS). (**a**) PCL-MWCNT nanofibers, (**b**) PCL-MWCNT nanofibers coated with CS, (**c**) PCL-MWCNT film, (**d**) PCL-MWCNT film coated with CS.

The Raman spectra of pristine PCL/MWCNTs nanofibers (a) and film (c) and the nanofibers coated with CS (b) and film coated with CS (d) are illustrated in Fig. 6. In the case of pristine PCL/MWCNTs nanofibers and film, two Raman bands appeared at 1351 and 1582 cm^–1^ which are attributed to D and G bands of MWCNTs, respectively (Zeng et al., 2006). These bands appeared stronger in intensity in the case of the film as compared to the fiber, indicating that the MWCNTs are mostly encapsulated inside PCL nanofibers. We assume that in this study there was minimal direct contact between the cells and carbon nanotubes. On other hand, new bands appeared at 1442, 1305, and 1090 cm^–1^ which are attributed to CH2 bending vibration, sulfate group asymmetric stretching.

### Biocompatibility studies

#### hDPSCs isolation and characterization

**Figure 7:**
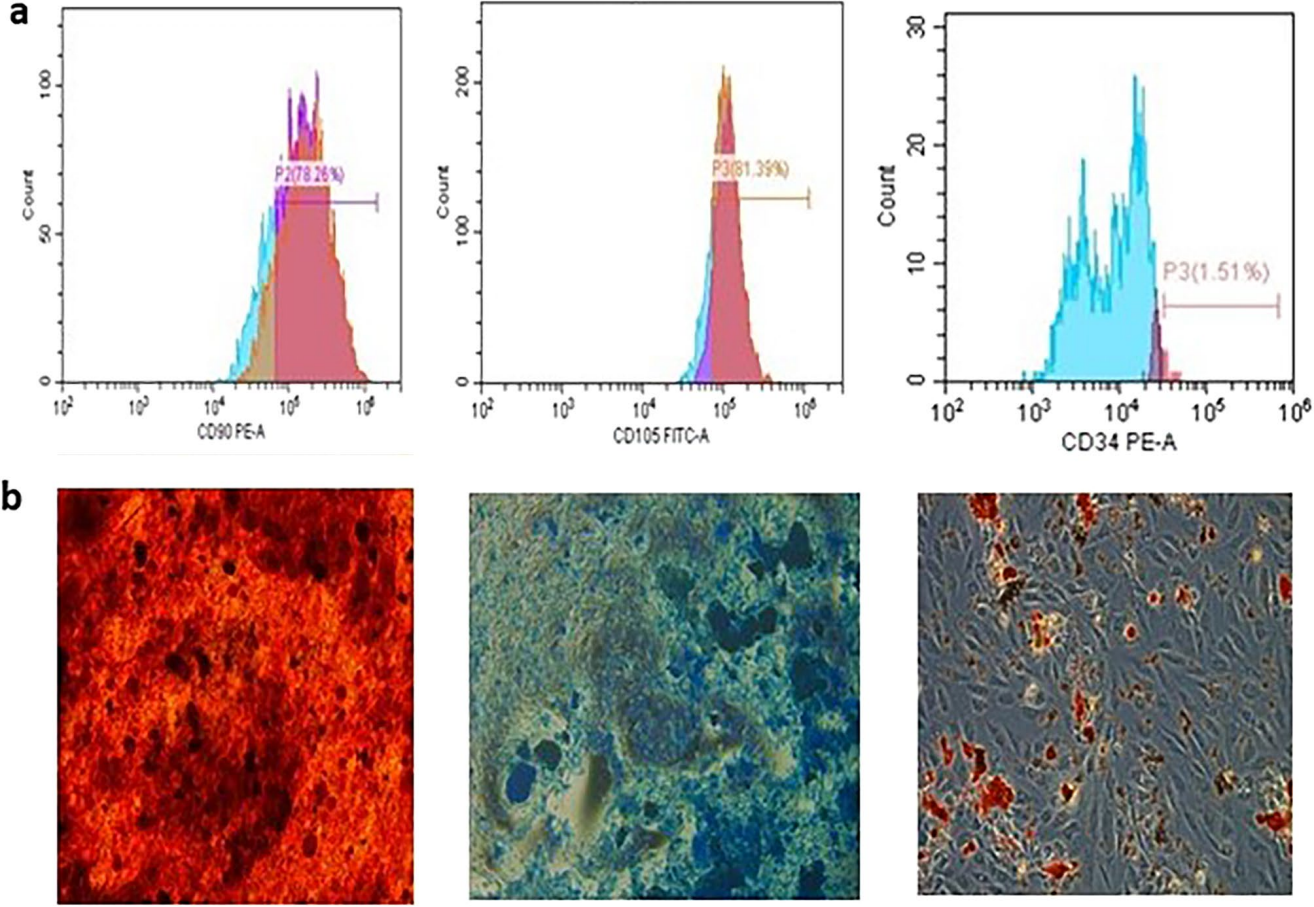
Characterization of isolated hDPSCs via flow cytometry (**a**) and multilineage differentiation potential (**b**) assessment. The isolated cells were positive for CD90 (78.6%) and CD105 (81.31%) and negative for CD34 (1.51%). The pluripotency of hDPSCs was assayed after culture in specific induction media for 21 days for osteogenic, chondrogenic and adipogenic differentiation. Calcific nodules were stained with Alizarin Red (left), and GAGs were stained with Alcian blue for chondrogenic differentiation (middle), while adipogenic differentiation was detected with Oil Red O staining of lipid droplets (right).

The isolated human dental pulp stem cells (hDPSCs) exhibited a characteristic fibroblast-like morphology and adhered to culture plates under conventional culture conditions. Flow cytometry, as depicted in Fig. 7a, revealed that the isolated hDPSCs exhibited positive expression of the MSC markers CD90 (78.26%) and CD105 (81.39%) but lacked expression of the hematopoietic stem cell marker CD34 (1.51%). The characterization results indicated that the isolated hDPSCs exhibited features consistent with those of MSCs, as defined by the International Society for Cellular Therapy (ISCT)^21^. Furthermore, hDPSCs multipotency was confirmed through the induction of adipogenic, chondrogenic, and osteogenic differentiation for a duration of 21 days (Fig. 7b). The production of lipid droplets was verified using Oil Red O staining, glycosaminoglycans (GAGs) were identified using Alcian blue staining, and calcium nodules were identified via Alizarin Red staining.

#### Cell viability and proliferation

**Figure 8:**
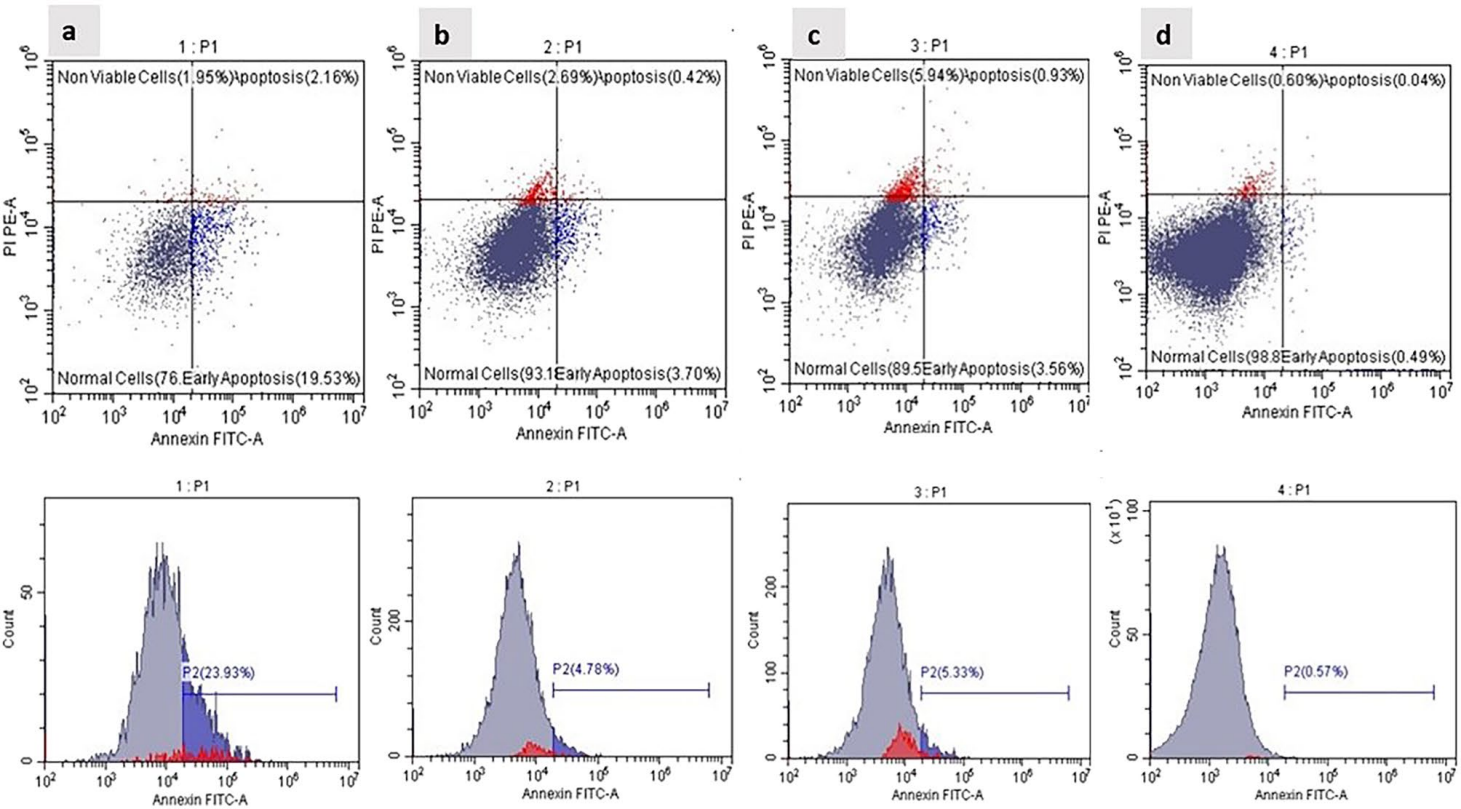
Flow cytometry analysis of Annexin V. (**a**) PCL-MWCNT/CS nanofiber, (**b**) PCL-MWCNT/CS film, (**c**) PCL-MWCNT nanofiber and (**d**) PCL-MWCNT film groups.

The possible cytotoxic effect of scaffold composition on cell viability and proliferation was observed through an Annexin V flow cytometric assay after 7 days. hDPSCs were seeded at a density of 1 × 10^4^ on PCL-MWCNTs films and nanofibers with and without CS coating and were allocated into 4 groups. Annexin V analysis (Fig. 8) revealed that the percentages of intact cells were 76.35 ± 0.39, 93.18 ± 0.45, 89.57 ± 0.49, and 98.87 ± 0.38% in the PCL-MWCNTs/CS nanofiber, PCL-MWCNTs/CS film, PCL-MWCNTs nanofiber, and PCL-MWCNTs film groups, respectively.

### hDPSCs morphology and adhesion behavior on PCL-MWCNT scaffolds

**Figure 9:**
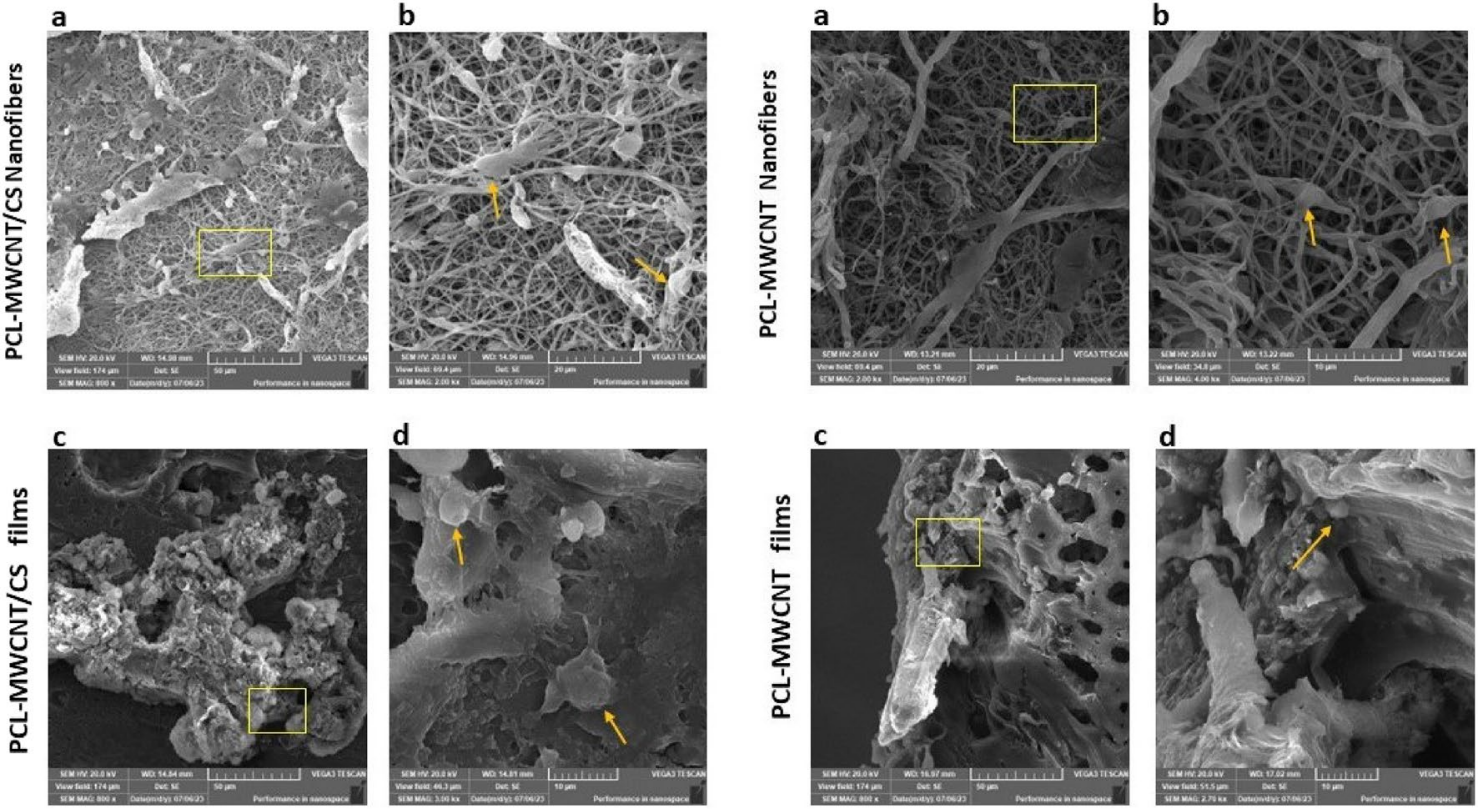
The morphology of hDPSCs cultured in DMEM on PCL-MWCNTs/CS nanofiber (**a**,**b**) and PCL-MWCNTs/CS films (**c**,**d**) scaffolds observed with scanning electron microscopy (SEM) at ×800 and ×2000–×4000 magnification. The square shows the magnification at ×2000–×4000, and the arrow indicates the cellular morphology.

**Figure 10:**
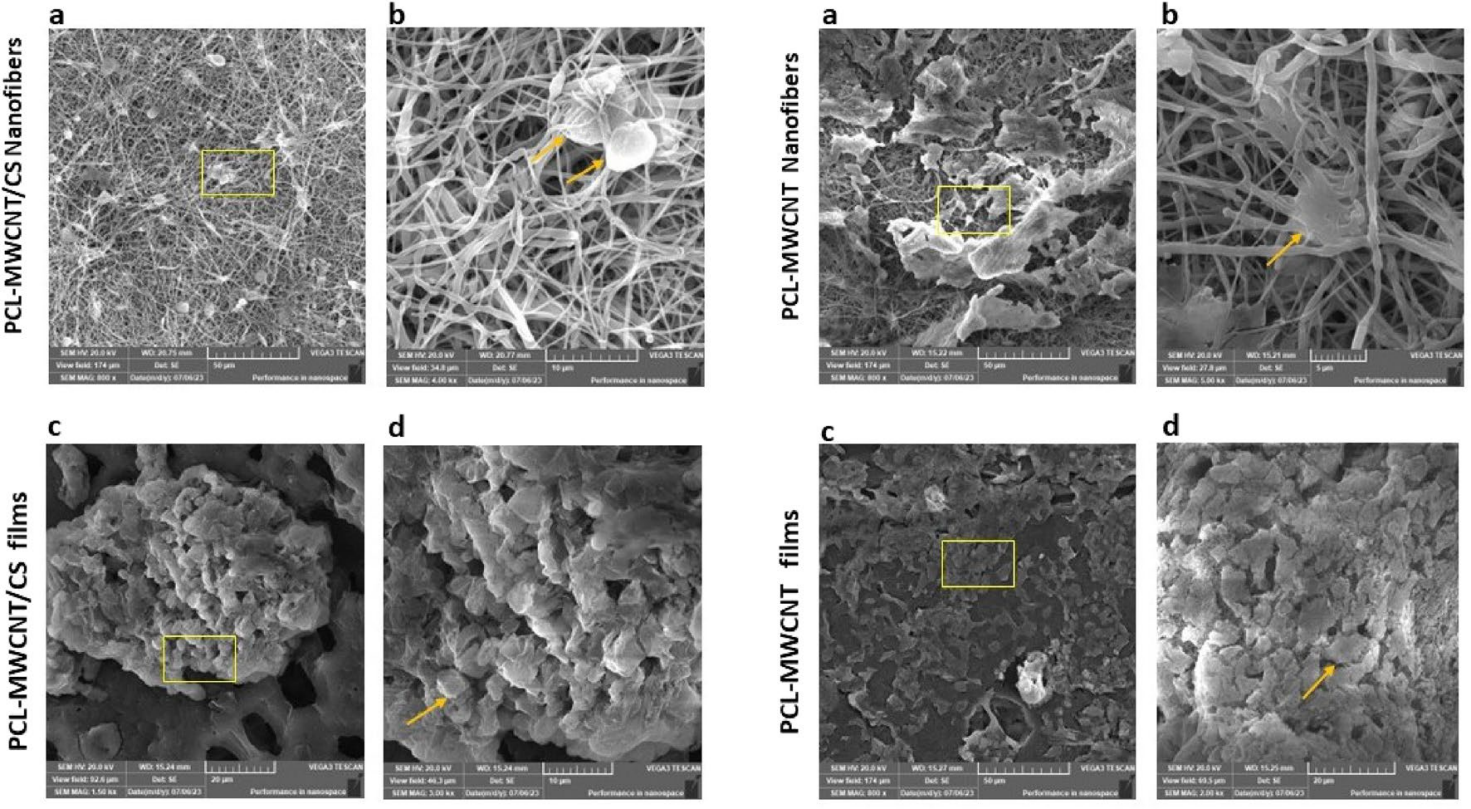
Morphology of hDPSCs grown on PCL-MWCNTs/CS nanofiber (**a**,**b**) and PCL-MWCNTs/CS films (**c**,**d**) scaffolds, cultured in chondrogenic media, observed with scanning electron microscopy (SEM) at ×800 and ×2000–×5000 magnification. The square shows the area magnified at ×2000–×4000, and the arrow indicates the cell morphology. Cells seeded on PCL/CNT/CS nanofibers showed potential changes in morphology when cultured in chondrogenic media, exhibiting a spheroid/polygonal cellular shape.

Scanning electron microscopy (SEM) was conducted to achieve a sufficient level of accuracy in evaluating the cellular attachment and distribution of hDPSCs, as well as their interactions with PCL-MWCNTs scaffolds when cultured either in DMEM or chondrogenic media. The spreading and attachment of the cells on all the fabricated scaffolds were observed. Figure 9 shows that hDPSCs cultured on PCL-MWCNTs nanofiber scaffolds were more oblong and spread evenly throughout the scaffold surface than cells grown on film scaffolds, which were round and attached to the scaffold surface in the form of clusters. When hDPSCs were cultured in chondrogenic media, hDPSCs exhibited signs of differentiation in which all the cells assumed a spheroidal or polygonal shape, which was more pronounced on the CS-coated PCL-MWCNTs nanofibers (Fig. 10).

### mRNA expression levels of chondrogenic differentiation-related genes in hDPSCs

**Figure 11:**
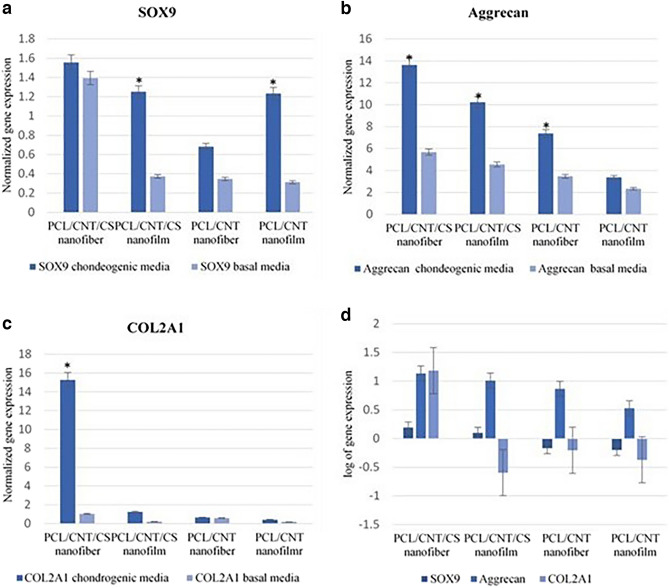
Log of relative gene expression of chondrogenic markers (SOX9, ACAN and COL2A1) in hDPSCs seeded on nanofibrous and film PCL-MWCNT scaffolds with and without chondroitin sulfate* (CS)*. (**a**) for SOX9, (**b**) for ACAN, (**c**) for COL2A1 and (**d**) shows the interpretation of the three genes expressed by cells seeded on different nanomaterials. Relative gene expression was normalized to cells grown on different nanomaterials in basal media. Results were the average of three independent experiments. The significance difference was calculated according to the *p*-value, (*) = *p* < 0.05.

**Figure 12:**
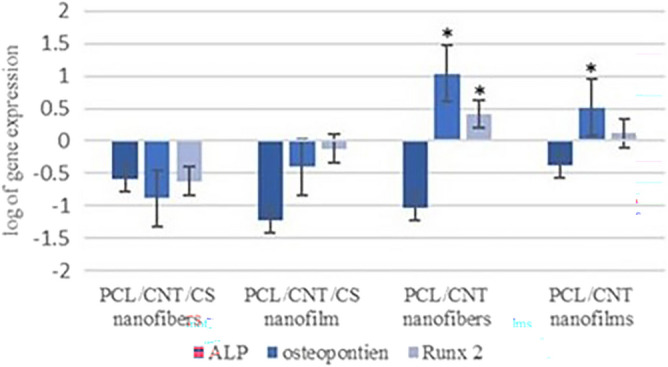
Log of the relative gene expression of osteogenic markers in hDPSCs seeded on nanofibrous and PCL-MWCNT film scaffolds with and without chondroitin sulfate (CS). Relative gene expression was normalized to undifferentiated DPSCs grown in basal media in plastic flasks for the same time. The significance difference was calculated according to the p-value, (*) = p < 0.05.

The expression of chondrogenesis genes related to hyaline-like cartilage, SOX9, Aggrecan and COL2A1, was assessed in all groups cultured both in chondrogenic and basal media for 21 days compared to the control group of cells that were cultured in ordinary cell culture flasks (Figs. 11, 12). The mean fold change for each target gene with the standard deviation (mean ± SD) and *p* value are listed in detail in Table 2. SOX9 was upregulated in cells seeded on PCL-MWCNTs nanofiber scaffolds with CS cultured in chondrogenic media, with a mean fold change of 1.55 ± 0.25, and in cells seeded in basal media, with a mean fold change of 1.39 ± 0.12, with no significant difference. Moreover, it was upregulated in cells seeded on PCL-MWCNTs films with CS coating, with a mean fold change of 1.25 ± 0.11 and a significant difference of 0.002 compared with that in cells cultured in basal media. SOX9 was downregulated in cells seeded on both PCL-MWCNTs nanofibers and films when they were cultured on either chondrogenic or basal media. Interestingly, Aggrecan was significantly upregulated in all the scaffolds cultured in both media for PCL-MWCNTs nanofibers, films with CS coating and PCL-MWCNTs nanofibers cultured in chondrogenic media. In chondrogenic induction media, Col2A1 was upregulated only in cells seeded on PCLMWCNTs nanofibers coated with CS, with a mean fold change of 15.28 ± 1.02 and a p value of 0.0008, and in cells seeded on films with a mean fold change of 1.25 ± 0.3 and a p value of 0.09. We also evaluated the expression of the osteogenesis-related genes RUNX2, ALP and OPN in hDPSCs cultured on the four fabricated scaffolds for 21 days in basal media. Relative gene expression was normalized to that of undifferentiated hDPSCs grown in basal media in plastic flasks. According to our results, the three osteogenic markers were not expressed in cells seeded on PCL-MWCNTs nanofibers or films with CS coating in comparison to control cells. Osteopontin was upregulated in cells seeded on both the PCL-MWCNTs nanofibers and films without the CS coating with mean fold changes of 10.95 ± 0.89 and 3.25 ± 1.01, respectively, with *p* values of 0.001 and 0.01, respectively. RUNX2 was also significantly upregulated in cells seeded on both PCL-MWCNTs nanofibers and films, with a mean fold change of 2.6 ± 1.2 (0.012) and 1.32 ± 0.98 (0.061), respectively.

**Table 2:** Mean fold change for each target gene with standard deviation (mean ± SD) and p value.

	SOX9			Aggrecan			COL2A1		
	Chondrogenic media (F. Ch.)	Basal media (F. Ch.)	*p*-value	Chondrogenic media (F. Ch.)	Basal media (F. Ch.)	*p*-value	Chondrogenic media (F. Ch.)	Basal media (F. Ch.)	p-value
PCL/CNT/CS nanofiber	1.55 ± 0.25	1.39 ± 0.12	0.138	13.6 ± 1.009	5.66 ± 0.98	0.006	15.28 ± 1.02	1.04 ± 0.03	0.0008
PCL/CNT/CS nanofilm	1.25 ± 0.11	0.37 ± 0.03	0.002	10.22 ± 0.44	4.54 ± 0.5	0.004	1.25 ± 0.3	0.23 ± 0.11	0.09
PCT/CNT nanofiber	0.68 ± 0.10	0.3 ± 0.22	0.156	7.37 ± 0.95	3.46 ± 0.90	0.0002	0.65 ± 0.2	0.58 ± 0.13	0.45
PCL/CNT nanofilm	0.63 ± 0.27	0.31 ± 0.13	0.17	3.37 ± 1.15	2.33 ± 0.96	0.098	0.42 ± 0.13	0.18 ± 0.1	0.02

## Discussion

Articular cartilage is a highly specialized type of connective tissue that covers the surfaces of bones involved in joint movement to provide smooth and frictionless articulation. However, when cartilage is damaged, its capacity for self-repair is limited, which often results in the degeneration of adjacent cartilage^22^. The objective of the present study was to fabricate a novel biocompatible scaffold intended for application in cartilage tissue engineering. The scaffold was designed to mimic the architecture of the extracellular matrix (ECM) and to create an optimal environment for cells to survive and differentiate, with the ultimate objective of restoring damaged tissues to their normal physiological function.

In the present study, polycaprolactone (PCL) was utilized to fabricate nanofibrous composite scaffolds by electrospinning. PCL is an important polymer in biomedical applications due to its biocompatibility, nontoxicity, and degradability, all of which are attributes that render it a suitable choice for scaffold fabrication^23^. Moreover, electrospinning is an ideal method for scaffold fabrication due to its ability to generate fibers at the nanoscale to microscale, which closely matches the structure of extracellular matrices (ECMs) observed in vivo^24,25^ and provides extensive flexibility in terms of morphological modification^26,27^. In the present study, to produce biocompatible nanocomposites that are effective for use in the regeneration of cartilage tissue, some modifications were implemented to the PCL scaffolds. First, we incorporated multiwalled carbon nanotubes (MWCNTs) as fillers into electrospun polycaprolactone (PCL). Although the utilization of MWCNTs as fillers in polymeric matrices is not widely practiced, it is acknowledged that they can be employed due to their exceptional mechanical and electrical characteristics^28–31^. The second modification involved the addition of a chondroitin sulfate coating to promote chondrogenic differentiation. Chondroitin sulfate was used as a component of the cartilage extracellular matrix to promote cartilage differentiation. For comparative analysis, four types of PCL-MWCNTs nanocomposites were used in either nanofiber or conventional film structures with/without chondroitin sulfate (CS).

Characterization of the nanofibrous and film architectures of the scaffolds was performed via FTIR and SEM. We utilized multiwalled carbon nanotubes (MWCNTs) in our study (d = 30–50 nm, purity > 95%) for the preparation of polymer-based scaffolds with 2.0% w/w MWCNTs. The electrospinning procedure successfully produced PCL-MWCNTs nanocomposites without any bead flaws, as revealed by our data. The fabricated PCL–MWCNTs nanofiber scaffolds exhibited increased porosity and a narrow range of diameters.

Cartilage is continually subjected to tensile stresses; thus, scaffolds for cartilaginous tissue regeneration applications must possess critical characteristics such as tensile strength to withstand stresses after insertion and to ensure successful healing when clinically applied^32^. Thus, enhancing the mechanical properties of the scaffolds is highly important. The Young’s modulus (rigidity) of the fibrous PCL-MWCNTs scaffolds was significantly greater than that of the film-structured scaffolds in the present study. This may be due to the fibrous structure, which includes porosity, and due to the rapid increase in the surface-to-bulk ratio of the fibrous structure.

One crucial characteristic of biomaterials is surface wettability, which influences cellular adhesion, migration, proliferation, and viability. A hydrophilic material is indicated by a contact angle less than 90°, while a hydrophobic material is indicated by a contact angle greater than 90°^33^. When the contact angle is less than 90°, the liquid wets the surface. When the contact angle is zero, wetting occurs^33,34^. We noticed that the addition of CS induced hydrophilic properties in both fibrous and film scaffolds, with a pronounced reduction in the contact angle. The presence of sulfate groups rendered the scaffolds hydrophilic. Furthermore, we observed that more water was immediately absorbed into the fibrous network than into the film structure, resulting in a decrease in the contact angle. Thus, it can be inferred that the integration of the fibrous structure along with the inclusion of CS resulted in a very hydrophilic effect and improved wettability. These characteristics create favorable conditions for promoting effective cellular adhesion. However, it is recommended to examine the incorporation of chondroitin sulfate at a lower concentration to adjust the scaffold to a moderate hydrophilicity level for achieving perfect adhesion.

Next, we assessed the biocompatibility of the fabricated scaffolds with human dental pulp stem cells (hDPSCs). Generally, the application of mesenchymal stem cells (MSCs) in the context of cartilage regeneration overcomes the drawbacks associated with unstable monolayer culture and the limited proliferative capacity of chondrocytes^35^. According to Anitua et al., dental pulp-derived stem cells (DPSCs) have demonstrated considerable potential as a cell type for tissue engineering for a number of reasons^36^. The accessibility of the surgical site is one of the reasons, as hDPSCs can be isolated from routinely extracted third molars and potentially retained for subsequent utilization while preventing additional harm to the donor site^37,38^. The hDPSCs isolated in the present study exhibited the distinctive attributes outlined by the International Society for Cellular Therapy (ISCT) as indicators of mesenchymal stem cells^21^. This was evident by the positive expression of CD90 (78.26%) and CD105 (81.39%) and negative expression of CD34 (1.51%), which was in agreement with previous reports^6,39^. In addition, the multipotency of the hDPSCs was confirmed by induction with adipogenic, chondrogenic, and osteogenic media for 21 days.

In the present study, the ability of the fabricated PCL-MWCNTs scaffolds to serve as bioactive scaffolds that could support the attachment, differentiation, and survival of hDPSCs was assessed. The fabricated PCL-MWCNTs nanofiber scaffolds demonstrated enhanced biocompatibility and the capacity to induce chondrogenic differentiation, rendering them highly promising for applications in cartilaginous tissue engineering. On the premise of this analysis, subsequent in vivo investigations into the treatment of articular damage can be conducted.

The biocompatibility of undifferentiated hDPSCs seeded on the different types of fabricated scaffolds was demonstrated via analysis of their prototype. Our results demonstrated that the film form of the PCL-MWCNTs enhanced the viability of the hDPSCs. This was confirmed by an Annexin V flow cytometric assay conducted on day 7, which demonstrated a significantly greater percentage of viable cells compared to the PCL-MWCNTs nanofibers. The morphology and attachment of the seeded cells were examined by SEM. The nanofibrous architecture of the PCL-MWCNTs promoted the attachment and distribution of hDPSCs, as observed by scanning electron microscopy. Scanning electron microscopy (SEM) images of seeded human dental pulp stem cells (hDPSCs) revealed a positive interaction with the PCL-MWCNT fibrous scaffold, as well as attachment to the surface through filopodia. The cells exhibited a fully extended morphology on the nanofibers, resembling a stellate shape. Scanning electron microscopy (SEM) images also revealed interactions between adjacent cells, which produced filopodia. We noticed that that the nanofilm scaffolds revealed more cellular proliferation, while the nanofibrous scaffold exhibited better cellular attachment. This behavior could be attributed to the presence of interconnected porosities in nanofiber matrices, which result from gaps between the fibers. The presence of these pores facilitates the attachment of cellular extensions, such as lamellipodia and filopodia, to the matrix. This results in a rapid transition of the attached cells from a circular shape to a flattened one. In the case of film scaffolds, cells may maintain their circular morphology during mitosis, resulting in an increase in cell count, in contrast to the flattened morphology that occupies a substantial surface area and diminishes cell count. Additional research is necessary to substantiate this hypothesis. Another possible factor could be the presence of MWCNT (multi-walled carbon nanotubes) on the surface of the film. Although most of the nanotubes were securely embedded within the nanofibers, their exposure could potentially affect the behavior of cells. The study by Lobo et al., revealed that the incorporation of multi-walled carbon nanotubes (MWCNT) facilitated the growth and multiplication of L-929 murine fibroblast cells^40^.

Finally, we assessed the chondrogenic differentiation induction capacity of the fabricated scaffolds. Our findings indicated that following chondrogenic induction for a period of 21 days, human dental pulp stem cells (hDPSCs) seeded on PCL-MWCNTs nanofibers and films with CS coating exhibited a noticeable transformation in shape. Specifically, they transitioned from an elongated stellate shape to a more polygonal shape, which is a strong indication of effective differentiation. Surface roughness of the scaffolds is also worth mentioning here since stem cells respond to the substrates through mechanotransduction pathways^41^. Although, there are huge difference between the surface roughness value of the nanofibers and film, the enhancement in cell adhesion and spreading on nanofibers might be due to the differences in topographical architecture and the presence of interconnected porosity in case of uncoated nanofibers. Whereas, for the coated fibers we believe surface chemistry plays a major role and the presence of familiar microenvironment for stem cells to differentiate into chondrocyte is a more important factor for enhancement of chondrogenic differentiation.

Subsequently, the analysis of the expression of chondrogenic genes, namely, SOX9, Aggrecan, and COL2A1, provided additional confirmation of the chondrogenic differentiation of human dental pulp stem cells (hDPSCs) seeded on the fabricated PCL-MWCNTs scaffolds. The expression of these genes was significantly elevated in hDPSCs seeded on PCL-MWCNTs/CS nanofibers. We observed this upregulation when the cells were cultured in both chondrogenic and basal media, with more pronounced expression in hDPSCs cultured in chondrogenic media. This might also be attributed to the rigid framework provided by the integration of nanofibers. Our molecular results were in agreement with those of previous studies that investigated the role of different bio scaffolds in promoting chondrogenesis in MSCs. Additionally, we evaluated the molecular expression of osteogenic-related markers in hDPSCs seeded with or without CS coating on PCL-MWCNTs scaffolds. Interestingly, the scaffolds without chondroitin sulfate (CS) coating showed an increase in the expression of markers related to osteogenesis, while this increase was not observed in the scaffolds with CS coating. This finding suggested that the PCL-MWCNTs may contribute to the initiation of spontaneous osteogenic differentiation.

Based on the findings from scanning electron microscopy (SEM) and the gene expression data, it could be concluded that both the nanofiber and film PCL-MWCNTs scaffolds with CS coating had the ability to enhance the chondrogenic differentiation of hDPSCs. These findings emphasized the significance of CS in promoting the chondrogenic differentiation of hDPSCs. Moreover, the rigidity of the nanofiber architecture could add value to the effect of CS in supporting differentiation, as chondrogenic gene markers were also upregulated even when cells were cultured in basal media. This finding was consistent with a previous study that assessed the use of scaffolds for chondrogenic induction of MSCs with and without chondroitin sulfate addition and reported that the inclusion of chondroitin sulfate in the fibers promoted the synthesis of type II collagen and improved the mechanical characteristics of tissues^42^.

In summary, our study demonstrated that the different forms of the fabricated PCL-MWCNTs scaffolds we analyzed demonstrated biocompatibility. Nevertheless, the nanofilm structures promoted a greater proportion of cell proliferation, while the nanofibrous architecture of the scaffolds enhanced the ability of the hDPSCs to attach and induce differentiation. Additionally, the effect of the CS coating on the biocompatibility and chondrogenic differentiation potential of hDPSCs was evaluated in the present study. Compared with the use of PCL-MWCNTs alone, chondroitin sulfate coating substantially enhanced the chondrogenic differentiation capacity of seeded hDPSCs. This observation indicated that chondroitin sulfate significantly contributed to the enhancement of the extracellular matrix of cartilage, as evidenced by the morphological change in the seeded cells from stellate and elongated to polygonal shapes, which was further confirmed by the expression of chondrogenic genes by hDPSCs.

## Conclusion

In conclusion, the results of the present investigation highlighted the significance of combining PCL with MWCNTs-incorporated nanofibrous together with CS coating on the viability, proliferation and chondrogenic differentiation capacity of hDPSCs This approach may be applied when designing PCL-based scaffolds for future cell-based therapeutic approaches developed for chondrogenic diseases.

## Materials and methods

### Synthesis of polycaprolactone-MWCNTs scaffolds

The polymer solution was prepared by dissolving PCL (10 wt%) in DCM/MeOH (3:1 v/v) upon stirring at room temperature adjusted from previous study^43^. MWCNTs were prepared by dispersing MWCNTs (2 wt%) in DCM/MeOH (3:1 v/v) via sonication (in a sonicator water bath) for 20 min at 25 °C. To form PCL-MWCNTs, PCL was gradually added dropwise to the MWCNTs dispersion and combined for 10 min at 25 °C in a sonicator water bath (Elmasonic S 180H, Germany) to create a homogenous combination. Using a 5 mL plastic syringe and an 18-gauge needle, the resulting matrix was subjected to electrospinning. There was a constant tip-to-collector distance of 10 cm, a 20 kV voltage, and a 0.5 mL/h flow rate. An electrically grounded piece of aluminum foil was used to cover the plate collector. The width of the spinnert was 40 mm, and its speed was adjusted to 100 rpm. Finally, the remaining solvent was removed from the nanofiber mats by drying them. The PCL-MWCNTs in the nanofibrous structure were compared to the PCL-MWCNTs in the form of films.

### Coating PCL-MWCNTs nanofibers and film with chondroitin sulphate (CS)

The coating of CS on PCL-MWCNTs nanofibers and film was conducted using NHS/EDC coupling agent similar to heparin in our recent study^44^. Briefly, after preparation of PCL-MWCNTs nanofibers and film, the material was cut with a scissor into squares (14 × 14 mm). The nanocomposites pieces were then aminolyzed with 10 wt% HMD dissolved in IPA for 3 h with moderate shaking at room temperature. After washing with deionized water, the pieces of nanofibers and films were treated with 25 mM EDC and 10 mM NHS dissolved in 50 mM MES buffer (pH = 5.5) to facilitate the coupling with the carboxylic groups of β-(1,4)-D-glucuronic acid structure of CS after carbodiimide activation. The pieces were placed in the bottom of 24 well plates and 2 ml of 10 mg/mL were added on each surface and moderately shacked for 24 h according to previous study^45^. The supernatant was decanted, and the samples were then washed with distilled eater and lyophilized using Christ bench top Freeze dryer (ALPHA model 1–2 LD plus, Germany) overnight.

### Characterization of polycaprolactone-MWCNTs scaffolds

#### Scanning electron microscopy (SEM) analysis

SEM was utilized to analyze the morphology of the nanofiber scaffolds compared to that of the films. The electrospun nanofibrous membranes were affixed to aluminum foil and then treated with a layer of gold palladium. SEM images were acquired 20 kV SEM (JEOL JSM-IT100, Japan), and the average fiber sizes were determined via the Image plus computer program.

#### Water contact angle of the fabricated scaffolds

The wettability of the fabricated scaffolds was assessed using the contact angle approach, following the ASTM D72499 and ASTM D594696 standard tests, with the assistance of Biolin Scientific contact angle analyzer (model T200, Sweden). The scaffolds were cut into square shapes measuring 3 × 3 cm^2^, and 5 μL of deionized water droplets were evenly distributed on their surface. The contact angles of both the fabricated nanofibrous and film PCL-MWCNT scaffolds were subsequently recorded after 10 s of adding the water drop and the image was captured and processed using device software.

#### Determination of mechanical properties

The Precision Universal Tester Autograph AGS-X Plus 20kN (Shimadzu, Japan) was used to study the mechanical characteristics, specifically Young's modulus, of the PCL-MWCNTs scaffolds in both nanofibrous and film architectures. The scaffolds were cut into rectangular 3 × 0.5 cm^2^ pieces and then securely fastened onto the machine jaws. The experiments were carried out at a stretching rate of 10 mm/min, and the collected data were recorded.

#### Surface roughness measurement

Gwyddion 2.32 software was chosen to calculate the average and root mean square roughness of uncoated and coated PCL/MWCNTs nanofibers and film from SEM micrographs similar to previous research with a little adjustment^46^. 10 different areas from 3 SEM images with a dimension of 30 × 30 μm (~ 778 × 775 pixels) for each square were selected for the roughness measurement.

#### Spectroscopic characterization

The chemical structures of the PCL-MWCNTs scaffolds were determined using infrared spectroscopy (EQUINOX 55, Bruker, Germany). Fourier transform infrared (FTIR) spectra were obtained by scanning in the spectral range of 500–4000 cm^1^. Furthermore, Raman spectroscopy was performed WITEC Alpha- 300 Raman confocal microscope (Germany) to evaluate the structural integrity of the PCL-MWCNTs scaffolds by analyzing the D and G bands and to examine the dispersion of the filler in the scaffolds.

### Isolation and expansion of hDPSCs

DPSCs were isolated from the pulp of third molars extracted from three patients who were presented for the extraction of impacted third molars at dental clinics affiliated with the National Research Centre. All patients provided informed consent prior to the extraction procedures. Approval No. 054180923 was granted by the Medical Ethics Research Committee (MERC) of the National Research Centre (NRC) for all experimental protocols. The hDPSCs were isolated as described by Gronthos (Gronthos, et al., 2002). Briefly, after extraction, the molars were temporarily stored in sterile DMEM containing 100 units/mL penicillin and 100 μg/mL streptomycin (pen/strep; Invitrogen). Subsequently, the molars were split open, and the dental pulp was excavated and cut into pieces of approximately 1 mm^3^. The pulp tissues were then digested using 0.5 mg/mL collagenase type I and incubated at 37 °C and 5% CO_2_ for 30 min. Single-cell suspensions were obtained using a 70 µm cell strainer. The cells were then cultured in DMEM supplemented with 10% FBS and 1% pen/strep at 37 °C and 5% CO_2_. The medium was gently replaced every three days after that. DPSCs were passaged at a density of 1500 cells/cm^2^ after reaching 70% confluency. Cells from the third passage of hDPSCs were used for all subsequent experiments^5,8^.

### Characterization of the isolated hDPSCs

#### Flow cytometric surface marker expression analysis

The isolated cell population was subjected to flow cytometric analysis (FACS) to verify the presence of mesenchymal stem cells (MSCs) and to eliminate the presence of non-MSCs. MSC markers were quantified using a Cytomics FC500 flow cytometer (Beckman Coulter, USA) along with CXP software. Briefly, the cells were detached and diluted to a concentration of 1 × 10^6^ cells/mL. After that, 1×106 cells were maintained in 10 mL of monoclonal antibodies targeting CD34 to rule out hematopoietic identity, as well as CD90 and CD105 to confirm the mesenchymal identity of the isolated cells at a temperature of 4 °C in the absence of light. Isotypes were used as a negative control. After being incubated for 20 min, tubes containing mAb-treated cells were administered 2 mL of PBS supplemented with 2% FBS. After centrifuging for 5 min at 2500 revolutions per minute (rpm), the supernatant was discarded, and the cells were resuspended in 500 millilitres (mL) of phosphate-buffered saline (PBS) containing 2% fetal bovine serum (FBS).

#### In vitro multilineage differentiation analysis

The multilineage differentiation potential of hDPSCs was evaluated using lineage-specific staining. Differentiation was achieved by seeding 5000 cells per well in 12-well culture plates. After the cells attached to the plates, the medium was changed to osteogenic, adipogenic, or chondrogenic differentiation medium. The cells were cultured for three weeks before evaluating their ability to differentiate successfully. hDPSCs were treated with a 0.3% solution of Oil Red O to detect the presence of lipid droplets outside the cells. Regarding chondrogenic differentiation, Alcian blue staining was utilized to identify the sulfated glucose amino glycan. To evaluate the capacity of hDPSCs to differentiate into osteogenic lineages, the cells were treated with a 1% solution of Alizarin Red S (Sigma‒Aldrich) for 20 min. The resulting mineralized nodules were then examined using an inverted light microscope (Leica, 6000B-4) equipped with Suite V3 software.

### DPSC seeding on scaffolds

Prior to the seeding of human dental pulp stem cells (hDPSCs) on the fabricated PCL/MWCNTs scaffolds, the scaffolds were sterilized for 60 min using 70% ethanol and UV radiation^47^. Then, the scaffolds were washed with PBS repeatedly and incubated at 37 °C for 24 h. In each of the twelve wells, 2.5 × 10^4^ hDPSCs were detached by trypsin and seeded onto the fabricated scaffolds both in films and nanofibrous structures with and without CS coating. The culture plates contained DMEM supplemented with 10% fetal bovine serum, 100 U/mL penicillin, 100 g/mL streptomycin, and 1 × amphotericin B. The plates were kept in an incubator at 37 °C with 5% CO_2_, and the medium was changed two to three times a week.

### Assessment of cell proliferation and viability of hDPSCs cultured on PCL-MWCNTs scaffolds

To quantitatively analyze the impact of the fabricated PCL-MWCNTs scaffolds on the survival and viability of hDPSCs, apoptotic cell labeling using an Annexin-V kit (Annexin/Alexa Fluor 488 and propidium iodide/PI—Thermo Fisher Scientific) was conducted on day 7 following cell seeding. hDPSCs seeded on nanofibrous and film-fabricated scaffolds were treated with trypsin followed by Annexin V reagent according to the manufacturer’s instructions^48^.

### Induction of chondrogenic differentiation of hDPSCs

hDPSCs from third-passage cultures were seeded on the four fabricated scaffolds at 2.5 × 10^5^ cells in StemPro^®^ chondrogenic differentiation medium (Invitrogen) for 21 days. The control hDPSCs seeded on the four fabricated scaffolds were cultured in complete DMEM without induction of differentiation.

### Electron microscopic evaluation of hDPSCs seeded on PCL-MWCNTs nanofiber and film scaffolds with CS coating

Scanning electron microscopy (SEM) was used to assess the cell morphology and behavior of hDPSCs on the fabricated scaffolds. After a period of 21 days of chondrogenic differentiation induction, hDPSCs seeded on the scaffolds were subjected to SEM morphological examination and compared to hDPSCs seeded on the scaffolds in full DMEM without chondrogenic induction. Briefly, PCL-MWCNTs nanofibers and films with or without CS coating were washed with PBS three times and then fixed with 3.5% glutaraldehyde for 1 h at 25 °C. Subsequently, the scaffolds were rinsed with PBS and dehydrated with ethanol. After an overnight air-drying process, the samples were examined via SEM. Subsequently, the scaffolds were rinsed with PBS and dehydrated in several percentages of ethanol (50, 70, 90, and 100%). After an overnight air-drying process, the samples were examined using SEM.

### Chondrogenic differentiation potential evaluation via qRT–PCR

Given that the purpose of the fabricated PC-MWCNTs is to enhance the production of cartilaginous tissue, it was essential to examine the behavior of hDPSCs on the variously prepared PCL-MWCNTs nanofibers and films with or without CS coating. To assess the chondrogenic differentiation ability, the cellular activity was examined in relation to the expression of chondrogenic genes in both chondrogenic and basal media after a 21-day culture period. Additionally, hDPSCs seeded on all types of fabricated scaffolds were cultured in basal DMEM and harvested after 21 days to assess the cellular behavior of the fabricated scaffold without induction of differentiation. Following a period of twenty-one days of chondrogenic induction of hDPSCs seeded on scaffolds, the total RNA of the cells was isolated from both induced and noninduced cells using Direct-zol RNA Microprep Kits (Zymo) according to the manufacturer’s instructions. RNA concentrations were determined by measuring the absorbance at 260 nm with a NanoDrop spectrophotometer (Fisher Scientific, Wilmington, DE, USA). A RevertAid First Strand cDNA Synthesis Kit (Thermo Scientific) was used, and 1 µg of extracted total RNA was used for cDNA synthesis. The synthesized cDNA was mixed with SYBR Green Master Mix. Gene expression was determined by real-time reverse transcription polymerase chain reaction (qRT‒PCR) in a Light Cycler 480 instrument (Roche) using the Light Cycler 480 Probes Master protocol. Amplification of mRNA was performed using custom primers for aggrecan (ACAN), sex-determining region Y-box 9 (SOX9), and collagen type-II (COL2A1). Moreover, osteogenic marker expression was also assessed for possible spontaneous osteogenic differentiation of seeded hDPSCs. The mRNA expression levels of the following osteogenic markers were investigated: runt-related transcription factor-2 (RUNX2), alkaline phosphate (ALP), and Osterix.

### Statistical analysis

Statistical analysis. There were three duplicates of each measurement. Using IBM SPSS software, version 20.0, t tests and ANOVA were used to examine the results, which are displayed as the mean ± standard deviation (SD). P values less than 0.05 were considered to indicate statistically significant differences between groups. One-way ANOVA and Post-hoc tests were used to assess the difference in contact angle among the various groups.

### Ethics declarations

The authors are accountable for all aspects of the work in ensuring that questions related to the accuracy or integrity of any part of the work are appropriately investigated and resolved. All methods were performed in accordance with the guidelines and regulations of the Declaration of Helsinki. Informed consent has been obtained from all participants. The experimental protocol was reviewed by the Ethical Committee of the Medical Research of the National Research Centre, Egypt and granted approval under the number 054180923.

## Data Availability

The datasets generated during and/or analyzed during the current study are available from the corresponding author on reasonable request.
